# Optimizing gentamicin dosing in different pediatric age groups using population pharmacokinetics and Monte Carlo simulation

**DOI:** 10.1186/s13052-021-01114-4

**Published:** 2021-08-06

**Authors:** Ragia H. Ghoneim, Abrar K. Thabit, Manar O. Lashkar, Ahmed S. Ali

**Affiliations:** 1 Pharmacy Practice Department, Faculty of Pharmacy, King Abdulaziz University, 7027 Abdullah Al-Sulaiman Rd, Jeddah, 22254-2265 Saudi Arabia; 2 Pharmacology Department, Faculty of Medicine, King Abdulaziz University, Jeddah, Saudi Arabia; 3 Department of Pharmaceutics, Faculty of Pharmacy, Assiut University, Assiut, Egypt

**Keywords:** Gentamicin, Pediatrics, Pharmacokinetics, Monte Carlo simulation

## Abstract

**Introduction:**

The use of once daily dosing of aminoglycosides in pediatrics is increasing but studies on dose optimization targeting the pediatric population are limited. This study aimed to derive a population pharmacokinetic model of gentamicin and apply it to design optimal dosing regimens in pediatrics.

**Methods:**

Population pharmacokinetics of gentamicin in pediatrics was described from a retrospective chart review of plasma gentamicin concentration data (peak/ trough levels) of pediatric patients (1 month − 12 years), admitted to non-critically ill pediatrics. Monte Carlo simulations were performed on the resulting pharmacokinetic model to assess the probability of achieving a C_max_/MIC target of 10 mg/L over a range of gentamicin MICs of 0.5–2 mg/L and once daily gentamicin dosing regimens. Results: A two-compartment model with additive residual error best described the model with weight incorporated as a significant covariate for both clearance and volume of distribution. Monte Carlo simulations demonstrated a good probability of target attainment even at a MIC of 2 mg/L, where neonates required doses of 6-7 mg/kg/day and older pediatrics required lower daily doses of 4–5 mg/kg/day while maintaining trough gentamicin concentration below the toxicity limit of 1 mg/L. Conclusion: Once daily dosing is a reasonable option in pediatrics that allows target attainment while maintaining trough gentamicin level below the limits of toxicity.

## Key points

1- Total body weight is a significant covariate that affects both clearance and volume of distribution of gentamicin in pediatrics.

2- Once-daily doses starting from 4 mg/kg/day provided adequate target attainment even at a MIC of 2 mg/L for chilldren.

3- Younger pediatrics require higher daily doses of gentamicin than older pediatrics but are complicated by reduced renal clearance.

## Introduction

Gentamicin is a bactericidal aminoglycoside antibiotic with potent activity against Gram-negative bacilli and synergistic activity with β-lactam antibiotics against Gram-positive cocci [1]. Gentamicin has been approved for use in the treatment of serious infections in all age groups, neonates to adults [1]. Nonetheless, the use of gentamicin is limited by nephrotoxicity and ototoxicity. One main strategy that has been used and proven to ensure maximal efficacy while mitigating the risk of toxicities of aminoglycosides, including gentamicin, is once daily dosing. This strategy is based on the pharmacokinetic (PK) and pharmacodynamic (PD) properties of gentamicin where a PK/PD index of free peak drug concentration above the minimum inhibitory concentration of the infecting organism (*f*C_max_/MIC) should be the focus of dosing [2]. The use of once daily dosing of aminoglycosides in pediatrics is increasing, especially in some disease states that require higher drug concentration due to increased drug clearance such as cystic fibrosis.

The pharmacokinetics in pediatrics are different than in adults. For example, neonates and infants have higher extracellular fluids per kilogram than children and adults. That would affect the volume of distribution of water-soluble medications, like aminoglycosides, resulting in a higher volume of distribution which decreases with age [3]. Renal elimination is also affected by age. Nephrogenesis is completed late in gestation; thus, premature neonates have compromised renal function. This reduction in glomerular filtration rate (GFR) affects renal drug clearance requiring longer dosing intervals in neonates. However, GFR increases with age and exceeds adult values during childhood but it gradually decreases to approximate adult values during adolescence [3].

Two recent studies have also evaluated gentamicin PK in pediatrics [4, 5]. The first study employed a one-compartment model to characterize gentamicin PK followed by Monte Carlo simulation to evaluate the optimal dosing for all patients aged 1 month to 12 years at different MICs. On the other hand, the second study only characterized the PK of gentamicin using a two-compartment model.

Monte Carlo simulation is a common method sought to compare and assess the probabilities of achieving different PK/PD targets (e.g., C_max_/MIC) using various dosing regimens [6, 7]. Results from these simulations are presented as the probability of target attainment (PTA) measured at different MICs. Eventually, such data can help guide optimal antibiotic dose selection; hence, fostering antimicrobial stewardship [8].

While several studies are available on gentamicin PK properties in the pediatric population, studies on dose optimization targeting this population are limited. And studies stratifying the recommendations to different pediatric groups are lacking. Therefore, this study aimed to provide recommendations on gentamicin once daily dosing in pediatrics of different age groups across a range of MICs using Monte Carlo simulation and assessment of PTA.

## Methods

### Patients and data

Plasma concentration data were obtained from a retrospective chart review of non-critically ill pediatric patients admitted to King Abdulaziz University Hospital, Jeddah, Saudi Arabia from February 2015 to November 2015. The study protocol was approved by the Biomedical Research Ethics Committee of the Faculty of Medicine, King Abdulaziz University, Jeddah, Saudi Arabia. Any patient aged from neonate to 12 years who received intravenous (IV) gentamicin for empiric treatment of Gram-negative infections, was included in the study. Patients admitted to the pediatric intensive care unit, patients taking gentamicin for surgical prophylaxis, and patients who received other nephrotoxic drugs were excluded. Patients data were collected from electronic medical records. Patients demographics were presented descriptively using numbers, percentages, and mean ± standard deviation.

### Gentamicin treatment and blood sampling

Gentamicin dosing was weight-based and was administered via IV route. Gentamicin peak concentrations were measured half an hour at the end of a 30-min infusion of the third dose for, whereas trough concentrations were drawn just before the fourth dose.

Homogenous particle enhanced turbidimetric inhibitory immunoassay technique (PETINIA, Dimension Clinical Chemistry System, Stream lab – Dade Behring, Inc., Erlangen, Germany) was used to measure gentamicin concentrations. The analysis was validated by running quality control samples as specified by the reagent manufacturer. The coefficient of variation was less than 5%. Renal function was assessed by measuring serum creatinine.

### Population pharmacokinetic model development and evaluation

The population pharmacokinetics of gentamicin was analyzed using non-linear mixed effect modeling (Phoenix® NLME version 8.2, Certara, L.P., Princeton, NJ, USA). A structural base model was first developed where a one- and two-compartment models were tested with different statistical residual errors for best-fit; including additive, multiplicative, and mixed error models. Base model selection was guided by the minimum objective function test including minus twice the Log Likelihood Function (Δ-2LL) and the Akaike Information Criterion (AIC), in addition to visual predictive checks of observed and predicted concentrations for each model run [9].

Following optimal base model selection, covariates including age, gender, serum creatinine, and body weight were examined as possible determinants of variability in pharmacokinetic estimates. The selection of covariates to add to the model was determined using a stepwise forward addition followed by a stepwise backward deletion approach [10]. Each covariate was first added to the model at a time and then removed from the model at a time. To include a covariate in the stepwise addition, a Δ-2LL change of 6.64 was used as a cutoff point (significant level of 0.01) and a Δ-2LL change of 10.83 was used as a cutoff point (significant level of 0.001) to keep the covariate in the model.

To evaluate the final model, a Bootstrap procedure (1000 bootstraps) was performed to check how robust our parameter estimates are for the model. Visual Predictive Checks were also carried out to determine the predictive power of the final model using 1000 simulated replicates of the original data sets, with the 5th, 50th^,^ and 95th quantile calculated for the simulated data and the observed data.

### Monte Carlo simulations

Monte Carlo technique (Phoenix NLME 8.2, Certara, L.P., Princeton, NJ, USA) was used to simulate peak and trough gentamicin concentrations following a 1-h infusion of gentamicin based on the final covariate model. 1000 simulations were performed on each of the four pediatric age groups as follows: Neonate (1 month), infants (2–12 months), toddlers (1–2 years), and children (5–10 years). These age groups were chosen based on pharmacokinetic differences in pediatrics across different age groups [11]. To simulate realistic demographic data, weight for each average age per group was based on the 50th percentile estimate of the typical weight of boys adopted from the Centers for Disease Control standard growth charts. Doses ranging from 2 to 8 mg/kg/day were used. The Probability of Target Attainment (PTA) was calculated using a C_max_/MIC target of 10 mg/L over a range of gentamicin MICs of 0.5–2 mg/L. This MICs range was selected because it falls below the susceptibility breakpoint of gentamicin against Gram-negative bacilli (Enterobacteriaceae, *Pseudomonas aeruginosa*, and *Acinetobacter baumannii*) according to Clinical Laboratory Standards Institute guidelines of 2020 [12]. The target was said to be attained if PTA at steady state was ≥90%. The target that was evaluated for safety was a C_min_ of < 1 mg/L.

## Results

### Patient demographics and data collection

**Fig. 1:**
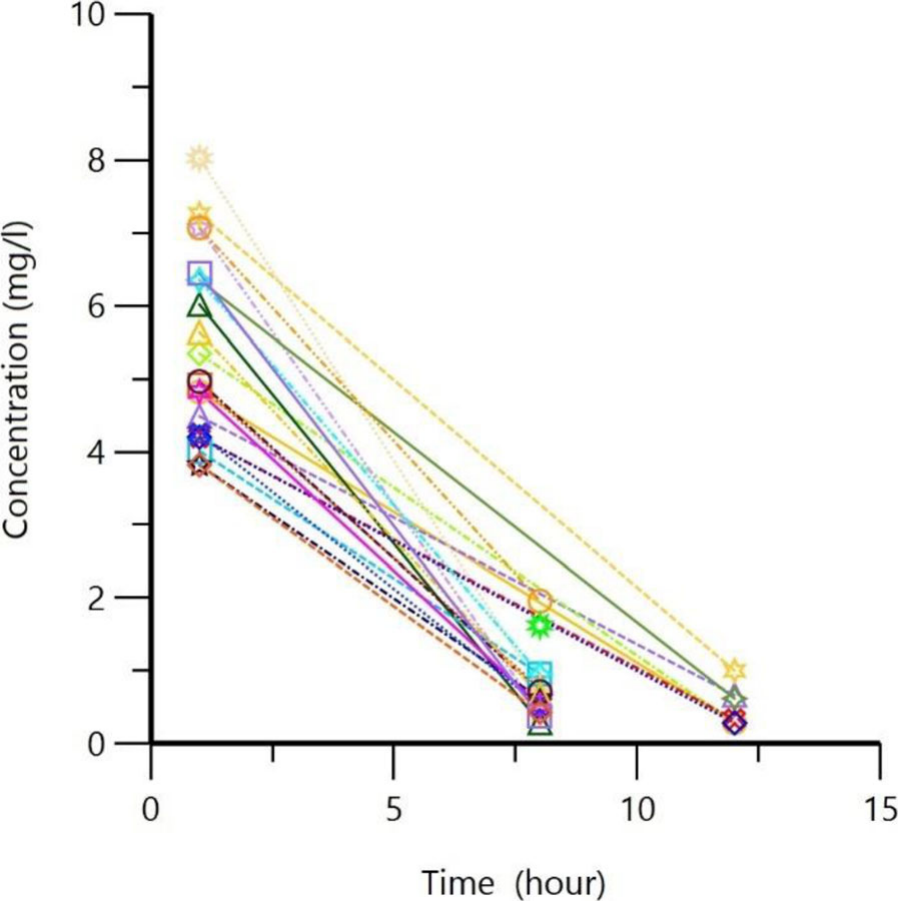
Observed gentamicin concentrations after IV infusion in pediatric patints (1 m-12 yr)

During the study period, 22 evaluable patients were identified and included. Table 1 lists the baseline characteristics of patients and gentamicin measurements. More than half of the patients were males and the mean age was approximately 3 years (34.88 ± 31.9 months). The mean dose was 2.75 ± 0.33 mg/kg with a median dosing interval of 8 h. Respective mean peak and trough concentrations were 5.45 ± 1.08 and 0.58 ± 0.28 mg/L, respectively (Fig. 1).

**Table 1:** Baseline patients demographics and gentamicin characteristics (*n* = 22)

Parameter	N (%) or mean ± SD (range)
Age (months)	34.88 ± 31.9 (1–72)
Sex (male)	13 (59.1)
Body weight (kg)	10.13 ± 5.25 (3.98–17.7)
Serum creatinine (mg/dL)	0.39 ± 0.82 (0.27–0.51)
Dose per weight (mg/kg/dose)	2.26 ± 0.33 (1.78–2.73)
Total daily dose (mg/kg)	22.75 ± 11.67 (10–40)
Dosing interval (hours)^a^	8 (8–12)
Peak concentration (mg/L)	5.45 ± 1.08
Trough concentration (mg/L)	0.58 ± 0.28

^a^ Data presented as median [interquartile range]

### Population pharmacokinetic model

**Fig. 2:**
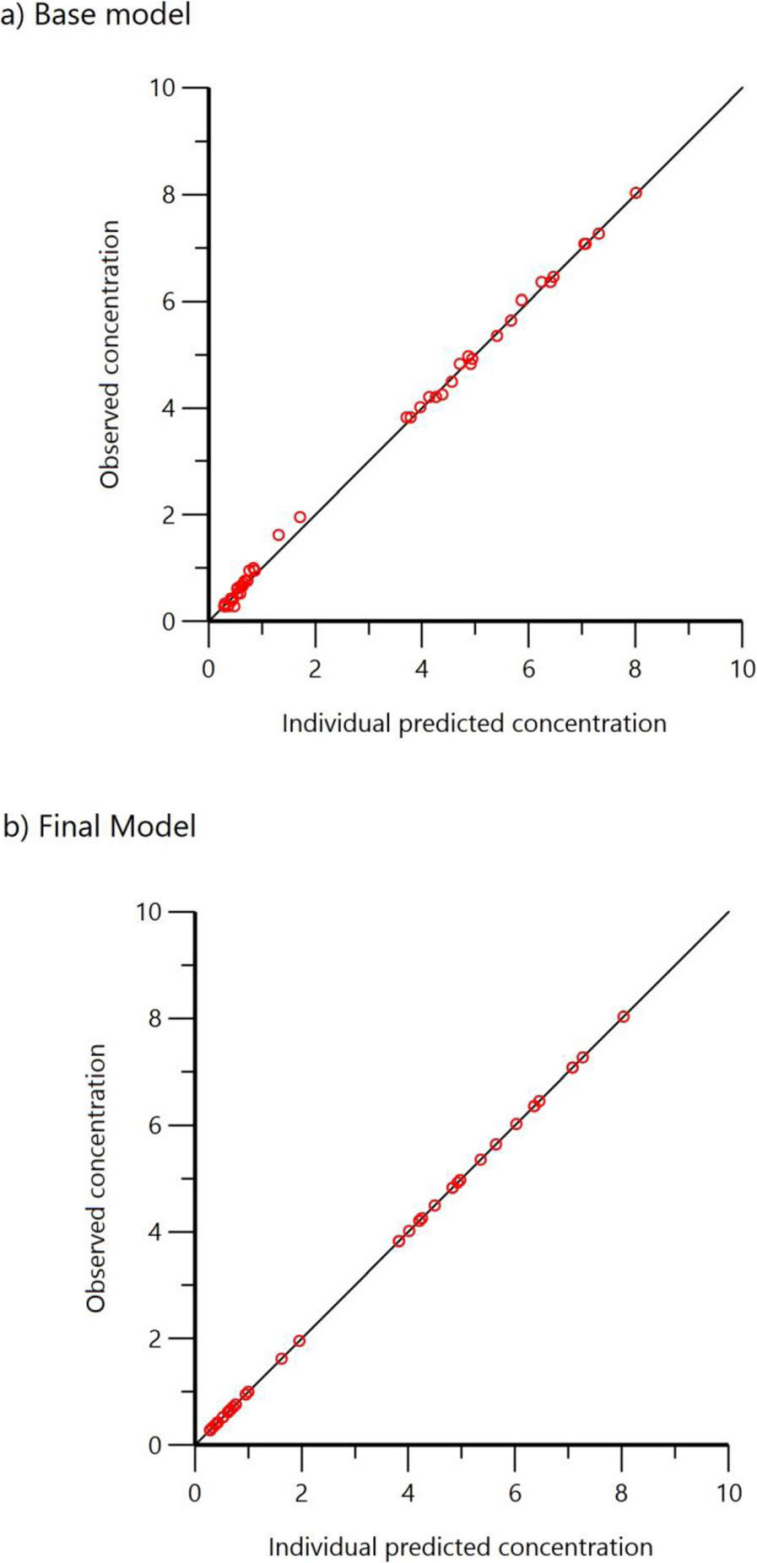
Diagnostic plots of base and final population PK model Observed and model predicted plasma gentamicin concentrations in base a) and final model b)

A two-compartment IV infusion model with additive residual error and first-order elimination best described the data in this patient population. Diagnostic plots of the base model showed a good fit with the minimal trend of residual error over time. PK parameters of the base model are shown in (Table 2). After establishing the base model, the effect of covariates on the PK parameters was investigated. This included continuous covariates, such as body weight, age, and serum creatinine, as well as categorical covariates, such as gender. Including the effect of weight on clearance and volume of distribution in the model improved the overall model by decreasing the Objective Function Value (OFV) -2LL by 61 points and AIC by 57 points, as well as decreasing the between-subject variability in clearance and volume of distribution. The parameter estimates of the final model and between subject variability are shown in (Table 2). Diagnostic plots of base and final models are shown in (Fig. 2). In the final model, clearance was estimated to be 4.64 L/hr./70 kg and was best described by the equation: 6 × (*weight in kg*/70)^0.71^. The average volume of the central compartment was 15.87 L/70 kg and best described by the equation: 15.87 × (*weight in kg*/70)^0.93^.

**Table 2:** Parameter estimate for the base model without the addition of covariates

Parameter	Mean estimate of the final model ± SE
Central volume (V_c_) (L)	2.01 ± 0.023
Peripheral colume (V_p_) (L)	3.32 ± 0.28
Clearance (CL) (L/h)	1.15 ± 0.022
Intercompartmenral clearance (Q) (L/h)	0.75 ± 0.044
Between subject variability associated with Vc (%)	105%
Between subject variability associated with CL (%)	60%
Shrinkage Eta Vd	0.22
Shrinkage Eta Cl	0.066
Additional error (mg/L)	0.28

### Model evaluation

**Fig. 3:**
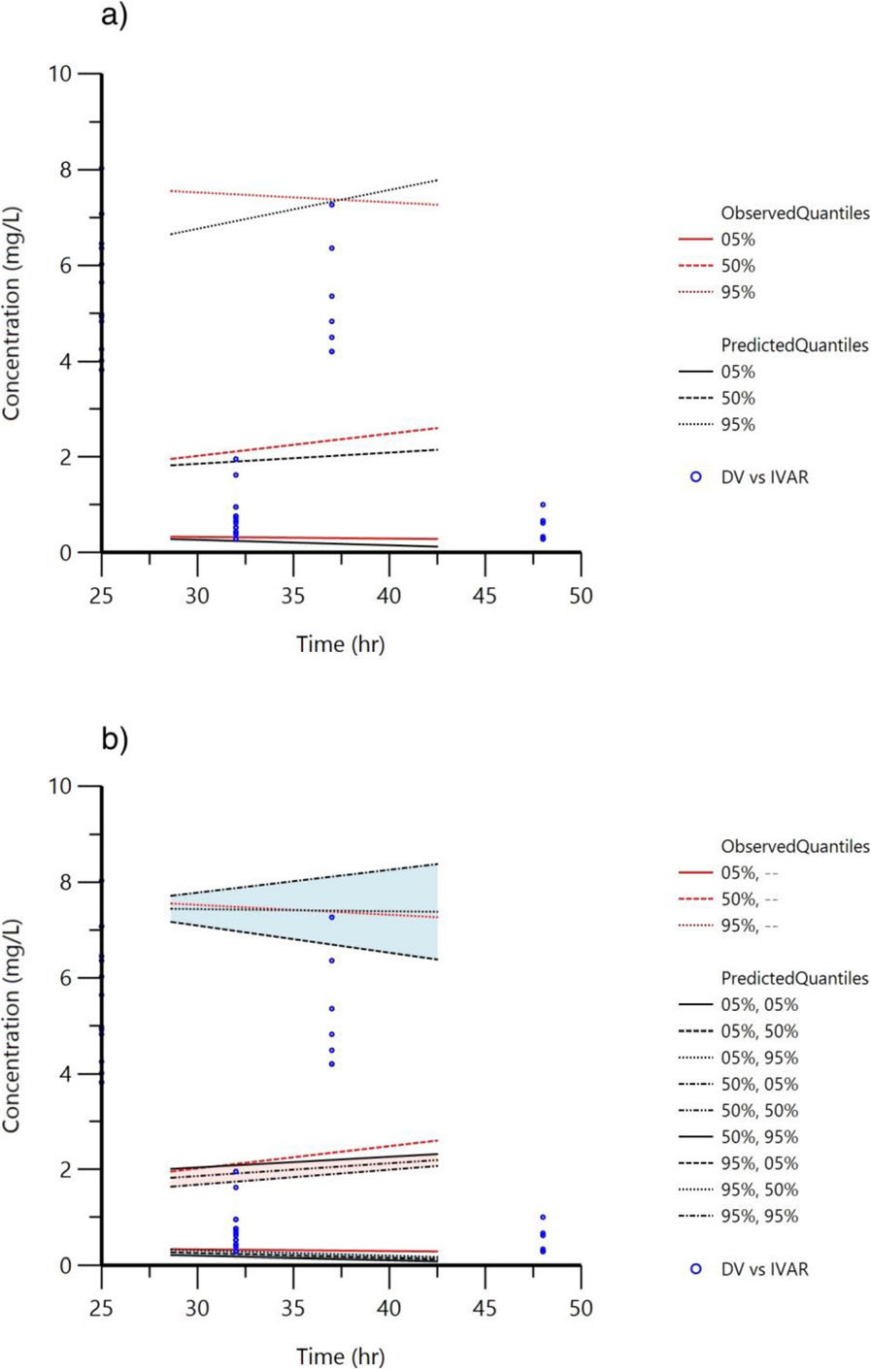
Visual predictive checks based on 1000 simulations of the final PopPK model. a) Graphical comparison of the 95, 50 and 5% quantiles observed and simulated (predicted) at each time point. b) The 95, 50 and 5% quantiles observed and simulated (predicted) at each time point and the 95, 50 and 5% confidence interval for each of the 3 quantile

The bootstrap demonstrated that the final model is adequate showing very similar final parameter estimates as compared with the final model estimates; the final model estimates were within the 95% of the bootstrap confidence interval and it does not appear to be sensitive to a particular subject within our samples population as seen in (Table 3). To determine the predictive power of the final model, visual predictive checks of the 5th, 50th^,^ and 95th percentiles demonstrated reasonable visual agreement between simulated and observed quantiles as shown in (Fig. 3).

**Table 3:** Parameter estimates for the final model and bootstrap

Parameter	Mean estimate of the final model ± SE	Bootstrap median result (95% confidence interval)
Central volume (V_c_) (L)	15.87 ± 3.99	15.86 (10.85–37.35)
Peripheral colume (V_p_) (L)	4.11 ± 0.99	4.11 (1.34–19.33)
Clearance (CL) (L/h)	4.64 ± 0.56	4.64 (3.58–7.34)
Intercompartmenral clearance (Q) (L/h)	0.62 ± 0.11	0.63(0.43–1.69)
Between subject variability associated with V_c_ (%)	37.80%	35.53%
Between subject variability associated with CL (%)	27.89%	27.00%
Additive error (mg/L)	0.011	0.011

### Monte Carlo simulation and the probability of target attainment

**Fig. 4:**
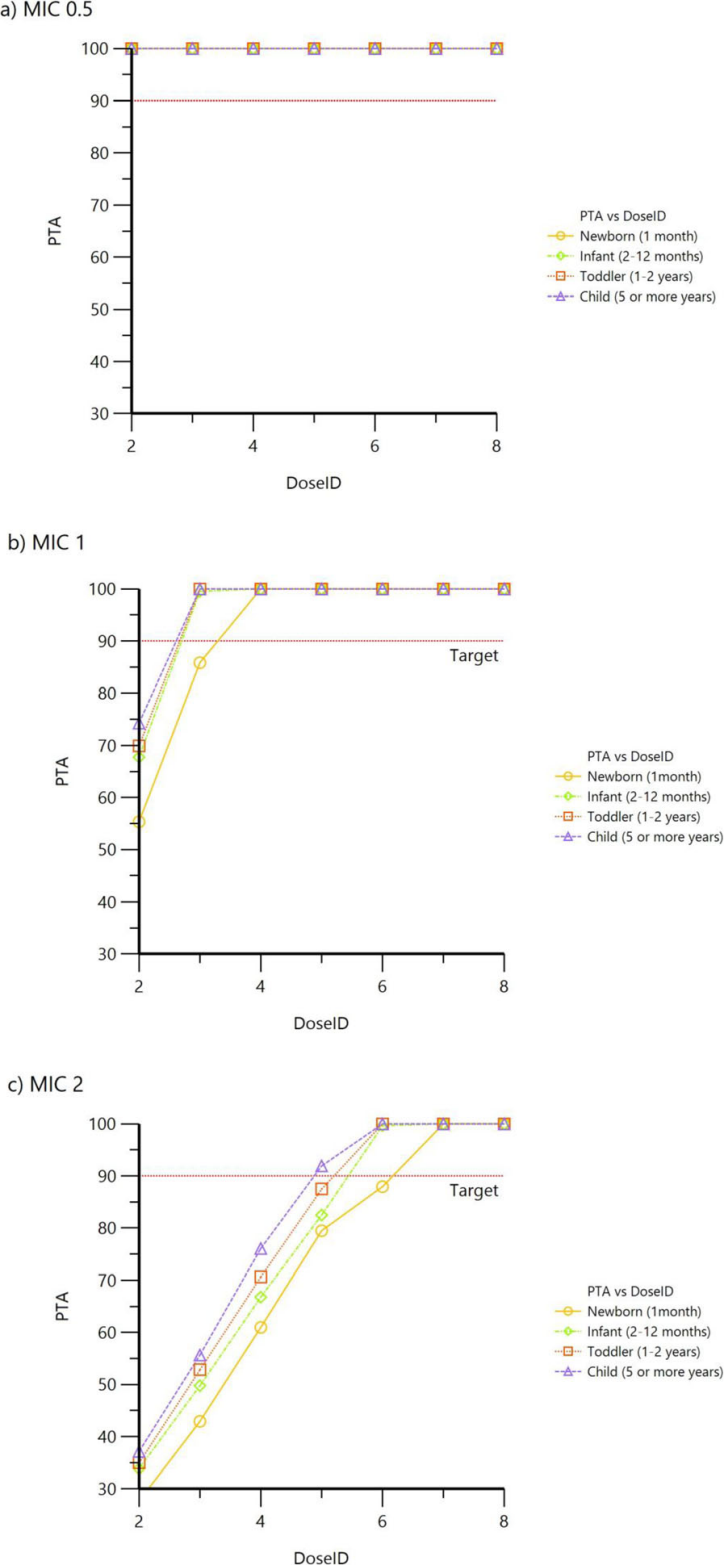
Target attainment analysis (PTA) for gentamicin for different age groups using various doses per kg (DoseID) and MIC values

**Fig. 5:**
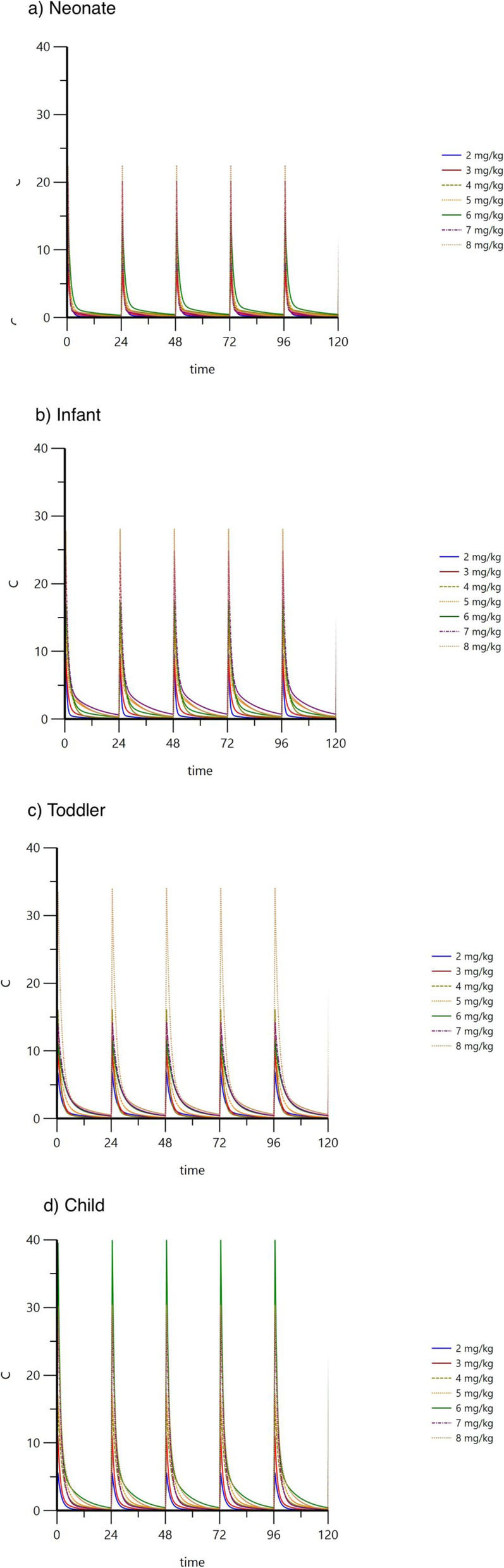
Simulation of predicted plasma concentrations© following various gentamicin dosing regimens in different age groups

Monte Carlo simulations were performed on 4 virtual groups of patients to estimate the > 90% PTA of C_max_/MIC of 10 or higher and a trough of < 1 mg/L using various once-daily dosing regimens at different MIC values (Fig. 4). At a MIC of 0.5 mg/L, all doses were able to achieve the therapeutic target. At a MIC of 1 mg/L, newborns only achieved the therapeutic target at doses of 4 mg/kg or higher, whereas doses of 3 mg/kg achieved targets in infants, toddlers, and children. At a MIC of 2 mg/L, newborns required doses of at least 6–7 mg/kg. Whereas doses of 5 and 4 mg/kg were sufficient in toddlers and children, respectively. Simulations of predicted concentrations with various doses are illustrated in (Fig. 5). Table 4 summarizes the recommended gentamicin doses for different pediatric age groups.

**Table 4:** Summary of daily dosing recommendations of gentamicin in different pediatric age groups

Age Group	Dose (mg/kg) every 24 h	
	MIC = 2 mg/L	MIC ≤ 1 mg/L
Neonate (1 month)	6–7	4–8
Infants (2–12 months)	6–7	4–8
Toddlers (1–2 years)	4–5	3–8
Children (5–10 years)	4–5	2.5–8

## Discussion

Despite the abundance of antibiotics, only a few are approved for use in the pediatric population, among which is gentamicin. Due to its broad-spectrum of coverage, it is often used empirically before the availability of culture and susceptibility results. It is also used as definitive therapy against susceptible organisms. However, due to the safety concern of nephrotoxicity, the dosing of aminoglycosides, including gentamicin, is based on body weight, as well as through monitoring. Previous studies have examined gentamicin PK and provided dosing recommendations in a particular age group or general recommendations for a wide age group range. Moreover, the rapid achievement of target peak and trough concentrations can ensure better outcomes. Therefore, this study comes to derive a population pharmacokinetic model of gentamicin and use it to identify optimal and safe dosing regimens in different pediatric age groups across a range of MICs below the susceptibility breakpoint of gentamicin against Gram-negative bacteria.

A two-compartment model with first-order elimination was used to describe the PK of gentamicin in our patient population, which produced a model with high precision and reliability to be used for simulations. This is in agreement with previous studies from a recent review, which included 33 studies on the gentamicin population PK in pediatrics up to 2017 and found that gentamicin PK in this population is mainly influenced by age, body size, and renal function [13]. Moreover, gentamicin PK can be altered by other factors, such as concurrent drugs, body temperature, and critical illness.

Most of the population PK studies of gentamicin in pediatrics focused on neonates and only a couple of studies included pediatric patients with age groups similar to our population [5, 11, 14, 15]. More recently, Wang et al. included physiological maturation of the extracellular weight into the model and estimated the values of clearance and central volume of distribution to be 4.6 L/hr. and 18 L, respectively. This is very similar to our final estimates of 4.6 L/hr. and 15 L despite using total body weight as a significant covariate. De cock et al. also included total body weight as a significant covariate for clearance and central volume of distribution [14]. However, oncology pediatric patients demonstrate augmented renal clearance and a higher final estimated clearance (5.77 L/hr./70 kg), as well as a central volume of distribution (21.6 L/70 kg) [15]. The study by Lopez et al. included critically ill patients who had lower estimates of clearance (2 L/hr. /70 k) and larger central volume (0.35 L/kg, which is around 25 L/70 kg) [11]. Indeed, gentamicin PK can be altered by other factors, such as concurrent drugs, body temperature, and critical illness. All these factors along with therapeutic drug monitoring should be considered when dosing and adjusting the dose of gentamicin in neonates and pediatrics.

In a recent study of 107 patients aged 1 month to 12 years where gentamicin PK was described using a one-compartment model, a Monte Carlo simulation showed that a dose of 5–6 mg/kg/day was adequate to treat infections caused by Gram-negative bacilli with a gentamicin MIC of ≤0.5 mg/L [4]. However, a higher dose of 7–8 mg/kg/day was needed when gentamicin MIC equals 1 mg/L. Both doses should achieve a C_max_/MIC of > 8 at a steady state. At MICs higher than 2 mg/L, gentamicin would not be recommended due to the low PTA where a 10 mg/kg/day dose achieved a PTA of only 52%. Our dose recommendations from this study apply to all age groups described herein. However, the difference lies in the stratification of the recommendations based on the MIC, whereas in our study, the recommendations were stratified based on different pediatric age groups in addition to MIC. Moreover, results from Monte Carlo simulations in our study demonstrated that lower doses than those indicated in previous reports might be sufficient. Dosing recommendations from our study were found to achieve PTA values of at least 90% when the MIC of gentamicin against the organism was 1 mg/L or lower while maintaining trough concentrations below the nephrotoxicity threshold of 1 mg/L, likely as a result of normal kidney functions in our population derived model. Consistent with our finding, a recent model that predicted dosing with a target to achieve a gentamicin C_max_ of 10 mg/L under a once daily dosing regimen suggested that dosing requirements decrease from birth (4 mg/kg) up to 18 years of age (2.7 mg/kg) [5]. Similar dosing recommendations were suggested by Lopez et al., despite including critically ill patients [11].

In cancer patients, a PK study showed that children who are 5 years old or younger continue to achieve low serum peak concentrations with once daily doses as high as 7 mg/kg compared to children older than 5 years [16]. This is likely a result of their higher clearance estimates. Hence, doses higher than the recommended here should be considered in this patient population with careful monitoring of the trough to ensure safety.

Pediatric pharmacokinetics is affected by age. The volume of distribution and GFR is highest during early childhood and then decreases to approximate adult values as the child grows into adolescence [3]. This was also suggested by our study, where the younger patients required higher doses per kilograms than older patients.

Our study was limited by a few factors. While kidney function in the pediatric population is typically assessed via measurement of urine creatinine, the retrospective nature of the study and the lack of documenting this information in the electronic medical records made it difficult to obtain such data. Also, we were limited by the lack of information on the post gestational age of newborns, which was documented as 4 weeks for all patients who were one month of age.

In conclusion, once daily dosing is a reasonable option for pediatrics at all age groups. Younger pediatric patients required higher weight adjusted daily doses than older children however, this may be complicated by reduced clearance.

## Data Availability

The datasets generated during and/or analyzed during the current study are available from the corresponding author on reasonable request.
